# Surgical and survival outcomes of lung cancer patients with intratumoral lung abscesses

**DOI:** 10.1186/s13019-017-0607-3

**Published:** 2017-05-26

**Authors:** Keiji Yamanashi, Norihito Okumura, Ayuko Takahashi, Takashi Nakashima, Tomoaki Matsuoka

**Affiliations:** Department of Thoracic Surgery, Kurashiki Central Hospital, 1-1-1 Miwa, Kurashiki, Okayama 710-8602 Japan

**Keywords:** Intratumoral lung abscesses, Lung cancer, Surgery

## Abstract

**Background:**

Intratumoral lung abscess is a secondary lung abscess that is considered to be fatal. Therefore, surgical procedures, although high-risk, have sometimes been performed for intratumoral lung abscesses. However, no studies have examined the surgical outcomes of non-small cell lung cancer patients with intratumoral lung abscesses. The aim of this study was to investigate the surgical and survival outcomes of non-small cell lung cancer patients with intratumoral lung abscesses.

**Methods:**

Eleven consecutive non-small cell lung cancer patients with intratumoral lung abscesses, who had undergone pulmonary resection at our institution between January 2007 and December 2015, were retrospectively analysed. The post-operative prognoses were investigated and prognostic factors were evaluated.

**Results:**

Ten of 11 patients were male and one patient was female. The median age was 64 (range, 52–80) years. Histopathologically, 4 patients had Stage IIA, 2 patients had Stage IIB, 2 patients had Stage IIIA, and 3 patients had Stage IV tumors. The median operative time was 346 min and the median amount of bleeding was 1327 mL. The post-operative morbidity and mortality rates were 63.6% and 0.0%, respectively. Recurrence of respiratory infections, including lung abscesses, was not observed in all patients. The median post-operative observation period was 16.1 (range, 1.3–114.5) months. The 5-year overall survival rate was 43.3%. No pre-operative, intra-operative, or post-operative prognostic factors were identified in the univariate analyses.

**Conclusion:**

Surgical procedures for advanced-stage non-small cell lung cancer patients with intratumoral lung abscesses, although high-risk, led to satisfactory post-operative mortality rates and acceptable prognoses.

## Background

Advanced-stage lung cancer often leads to respiratory infections, including intratumoral lung abscesses, post-obstructive pneumonia, or empyema. The prognosis of lung cancer patients with these respiratory infections is sometimes determined by these infections regardless of lung cancer progression. Specifically, intratumoral lung abscess is considered a fatal condition because it worsens the patient’s condition, interferes with the treatment of the primary tumor, and rupture of the lung abscess can cause empyema [1–6]. Therefore, surgical procedures for intratumoral lung abscesses have sometimes been performed [7]. However, these are considered high-risk due to (1) severe fibrosis adhesion to adjacent organ systems, (2) intra-operative rupture of the intratumoral lung abscess by peeling adhesion, (3) poor intra-operative vision associated with the intratumoral lung abscess, or (4) the poor general condition of the patients.

Few reports have been published concerning surgical procedures for non-small cell lung cancer (NSCLC) patients with respiratory infections. Recently, Haraguchi et al. [8] reported on the surgical outcomes (e.g., post-obstructive pneumonia) of NSCLC patients with respiratory infections. However, there have been no studies examining the surgical outcomes of NSCLC patients with intratumoral lung abscesses. Therefore, we conducted a retrospective cohort study to investigate the surgical and survival outcomes of NSCLC patients with intratumoral lung abscesses.

## Methods

### Patient selection

We conducted a retrospective cohort study of 11 consecutive NSCLC patients with intratumoral lung abscesses who were referred to our hospital for pulmonary resection between January 2007 and December 2015. The criteria for diagnosing intratumoral lung abscesses were as follows: (1) a lung tumor with a pre-operative pathological diagnosis of NSCLC and (2) lung tissue necrosis with the formation of cavities containing necrotic debris or fluid caused by a microbial infection in the tumor that is detected by computed tomography imaging or pathological evaluation. Radical pulmonary resection with mediastinal lymphadenectomy was performed for lung cancer except Stage IV disease and pulmonary resection including intratumoral lung abscesses in Stage IV disease.

The study protocol was granted approval by the appropriate Ethical Review Board committee of our institution. The requirement for patient informed consent was waived due to the retrospective nature of the study. Research was conducted in accordance with the 1964 Declaration of Helsinki and its later amendments.

### Evaluation of clinicopathological factors

The following clinical characteristics were retrieved from the available clinical records: age, sex, body mass index, Brinkman index, serum albumin levels, haemoglobin levels, white blood cell counts, C-reactive protein levels, percentage predicted vital capacity, percentage predicted forced expiratory volume in 1 s (FEV_1_), FEV_1_/forced vital capacity ratio, pre-operative performance status (PS), Charlson comorbidity index, presence or absence of empyema, explanation for the intratumoral lung abscess, tumor location and size, abscess and necrosis size, pathological stage and histological subtype of the lung cancer, surgical procedure, combined resection, operative time, extent of bleeding, and post-operative morbidity and mortality. Post-operative morbidity was defined according to the Common Terminology Criteria for Adverse Events, version 4.0 as complications occurring within 30 days after surgery. Post-operative mortality was defined as death occurring within 30 days after surgery. Overall survival (OS) was measured from the date of surgery to the date of death from any cause or last follow-up.

### Statistical analyses

OS curves were plotted using Kaplan-Meier method. Univariate analyses of OS outcomes were performed using a Cox proportional hazards model. All statistical data were processed and analysed using the statistical software R, version 3.0.3 (R Foundation for Statistical Computing, Vienna, Austria). All *P*-values were two-sided and a *P* < 0.05 was considered statistically significant.

## Results

### Subjects

Data from 11 consecutive NSCLC patients with intratumoral lung abscesses who had undergone pulmonary resection at our hospital between January 2007 and December 2015 were obtained from the hospital’s database. The patients’ clinicopathological characteristics are summarised in Tables 1 and 2. Ten of the 11 patients were male and one patient was female. The median age was 64 (range, 52–80) years. The median body mass index, Brinkman index, serum albumin levels, haemoglobin levels, white blood cell counts, C-reactive protein levels, percentage predicted vital capacity, percentage predicted FEV_1_, FEV_1_/forced vital capacity ratio, tumor size, abscess and necrosis size, operative time, and amount of bleeding were 22.3 (range, 19.4–24.7) kg/m^2^, 920 (range, 480–2400), 2.3 (range, 1.4–3.3) g/dL, 10.1 (range, 8.0–15.8) g/dL, 17.0 (range, 5.8–20.7) 10^3^/μL, 21.8 (range, 1.7–34.1) mg/dL, 101.7% (range, 44.5–131.1%), 81.9% (range, 33.2–112.2%), 66.5% (range, 45.0–81.9%), 55 (range, 31–107) mm, 55 (range, 25–96) mm, 346 (range, 222–456) minutes, and 1327 (range, 63–3134) mL, respectively. The proportions of patients with a pre-operative PS of 0–1 and a Charlson comorbidity index of 0–2 were 63.6% and 81.8%, respectively. Four patients experienced empyema. Intratumoral lung abscesses were developed after bronchoscopic biopsy in 8 patients, after computed tomography-guided biopsy in one patient, with bronchial obstruction in one patient, and uncertainly in one patient. The tumor was located in the right upper lobe in 4 patients, right lower lobe in 4 patients, left upper lobe in 2 patients, and left lower lobe in one patient. Histopathologically, 4 patients had Stage IIA, 2 patients had Stage IIB, 2 patients had Stage IIIA, and 3 patients had Stage IV tumours (additional nodules in the contralateral lung [*n* = 2] and brain metastasis [*n* = 1]). Five patients had adenocarcinoma, 4 patients had squamous cell carcinoma, one patient had large cell neuroendocrine carcinoma, and one patient had spindle cell carcinoma. Open thoracotomy was performed in 10 patients and video-assisted thoracoscopic surgery was performed in one patient. Lobectomy was performed in 9 patients, bilobectomy in one patient, and pneumonectomy in one patient. Combined resection was performed in 4 patients (parietal pleura [*n* = 2], mediastinal pleura [*n* = 1], and chest wall [*n* = 1]). The post-operative morbidity and mortality rates were 63.6% and 0.0%, respectively. Recurrence of respiratory infections, including lung abscesses, was not observed in all patients.

**Table 1:** Pre-operative characteristics of non-small cell lung cancer patients with intratumoral lung abscesses (*n* = 11)

No.	Age (y)	Sex	BMI (kg/m^2^)	BI	Alb (g/dL)	Hb (g/dL)	WBC (10^3^/μL)	CRP (mg/dL)	VC^a^ (%)	FEV_1_^a^ (%)	FEV_1_/FVC (%)	PS	CCI	E	Reason for lung abscess
1	63	M	22.3	860	3.7	9.5	7.2	2.8	124.1	103.0	64.5	0	3	–	BS biopsy
2	64	M	20.3	920	2.9	16.0	13.1	13.6	131.1	109.9	68.4	2	1	–	CT-guided biopsy
3	52	M	19.4	620	2.5	9.7	20.7	9.4	97.3	87.5	69.7	1	1	–	BS biopsy
4	64	M	21.0	1960	2.8	12.2	7.0	1.1	95.2	71.0	59.9	1	2	+	BS biopsy
5	80	M	24.3	2400	2.2	10.0	17.3	6.8	100.7	76.2	56.5	3	4	+	BS biopsy
6	67	M	22.5	840	2.3	10.1	6.8	6.8	102.7	93.6	72.7	1	0	–	BS biopsy
7	56	M	20.8	1140	3.7	12.4	5.8	0.3	114.2	112.2	81.9	0	0	–	BS biopsy
8	68	F	24.3	500	3.6	11.5	9.2	1.4	121.4	73.3	45.0	0	1	–	Unknown
9	64	M	24.7	1500	1.4	8.7	16.0	14.2	90.6	67.1	59.7	2	1	–	BO
10	73	M	21.8	1920	1.7	11.2	14.6	13.8	44.5	33.2	58.2	2	2	+	BS biopsy
11	65	M	23.5	480	1.7	8.8	14.6	9.6	86.2	74.3	69.2	1	1	+	BS biopsy

^a^Predicted values

*Abbreviations*: *Alb* albumin, *BI* Brinkman index, *BMI* body mass index, *BO* bronchial obstruction, *BS* bronchoscopy, *CCI* Charlson comorbidity index, *CRP* C-reactive protein, *CT* computed tomography, *E* empyema, *F* female, *FEV*_*1*_ forced expiratory volume in 1 s, *FVC* forced vital capacity, *Hb* haemoglobin, *M* male, *No.* number, *PS* performance status, *VC* vital capacity, *WBC* white blood cell

**Table 2:** Intra-operative and post-operative characteristics of non-small cell lung cancer patients with intratumoral lung abscesses (*n* = 11)

No.	Tumor location	Tumor size (mm)	Abscess size (mm)	Stage	Histology	Surgery (procedure)	CR	OT (mins)	Bleeding (mL)	Post-operative mobidity^a^	Survival (months)	Cause of death
1	LUL	75	68	IIIA	ADC	L (O)	–	322	210	Recurrent LNP (G3)	114.5 (A)	–
2	RUL	55	54	IV	ADC	L (O)	–	415	3134	SVA (G2)	27.7 (D)	LC
3	RUL	90	80	IV	SqCC	L (O)	–	456	2196	–	1.3 (D)	LC
4	LLL	51	50	IIA	ADC	L (O)	MP	349	1327	–	57.3 (A)	–
5	RLL	35	34	IIA	SqCC	L (O)	–	274	644	CI (G2)	16.1 (D)	LC
6	RLL	36	35	IIA	SCC	L (O)	–	222	170	–	35.7 (A)	–
7	RUL	31	25	IIB	ADC	L (V)	PP	235	75	–	16.4 (A)	–
8	LUL	60	55	IIA	SqCC	L (O)	CW	323	63	SCA (G2)	11.1 (A)	–
9	RUL	55	92^b^	IV	SqCC	P (O)	PP	347	1930	SVA (G2)	8.57 (A)	–
10	RLL	107	96	IIB	LCNEC	L (O)	–	346	1369	Hypoxemia (G2)	11.4 (D)	LC
11	RLL	75	74	IIIA	ADC	B (O)	–	421	1450	BPF (G4)	9.2 (D)	Pneumonia

^a^Common Terminology Criteria for Adverse Events, version 4.0

^b^Size of abscess and necrosis of whole lung lobe

*Abbreviations*: *A* alive, *ADC* adenocarcinoma, *B* bilobectomy, *BPF* bronchopleural fistula, *CI* cerebral infarction, *CR* combined resection, *CW* chest wall, *D* deceased, *G* grade, *L* lobectomy, *LC* lung cancer, *LCNEC* large cell neuroendocrine carcinoma, *LLL* left lower lobe, *LNP* laryngeal nerve paresis, *LUL* left upper lobe, *MP* mediastinal pleura, *No.*, number *O* open thoracotomy, *OT*, operative time, *P* pneumonectomy, *PP* parietal pleura, *RLL* right lower lobe, *RUL* right upper lobe, *SCA* subcutaneous abscess, *SCC* spindle cell carcinoma, *SqCC* squamous cell carcinoma, *SVA* supraventricular arrhythmia, *V* video-assisted thoracoscopic surgery

**Fig. 1:**
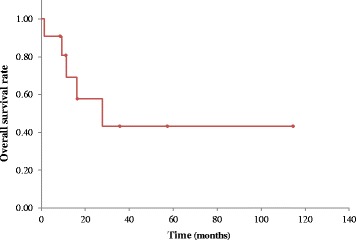
Kaplan-Meier curve of overall survival for non-small cell lung cancer patients (*n* = 11) with intratumoral lung abscesses

The median post-operative observation period was 16.1 (range, 1.3–114.5) months and the 5-year OS rate was 43.3% (Fig. 1).

### Univariate analyses of factors associated with overall survival

For statistical analyses of prognostic factors for OS, patients were stratified according to the following parameters: age (above or below the median of 64 years), sex (male *vs.* female), body mass index (above or below the median of 22.3 kg/m^2^), serum albumin levels (above or below the median of 2.3 g/dL), haemoglobin levels (above or below the median of 10.1 g/dL), white blood cell count (above or below the median of 17.0 × 10^3^/μL), C-reactive protein levels (above or below the median of 21.8 mg/dL), percentage predicted vital capacity (above or below the median of 101.7%), FEV_1_/forced vital capacity ratio (above or below the median of 66.5%), pre-operative PS (0–1 *vs.* 2–3), Charlson comorbidity index (0–2 *vs.* ≥3), presence or absence of empyema, tumor size (above or below the median of 55 mm), pathological stage (Stage II *vs.* Stage III–IV), histological subtype (adenocarcinoma *vs.* other), surgical procedure (open thoracotomy *vs.* video-assisted thoracoscopic surgery), surgery (lobectomy *vs.* other), combined resection, operative time (above or below the median of 346 min), and extent of bleeding (above or below the median of 1327 mL). The univariate analyses of OS outcomes using a Cox proportional hazards model revealed that there were no pre-operative, intra-operative, or post-operative prognostic factors (Table 3).

**Table 3:** Univariate analyses for overall survival

Variable		Patients (*n* = 11)	HR	95% CI	*P*-value
Age, years	<64	3	1.504	0.167–13.540	0.716
	≥64	8			
Sex	F	1	-	-	-
	M	10			
BMI, kg/m^2^	<22.3	5	0.649	0.108–3.910	0.637
	≥22.3	6			
Alb, g/dL	≥2.3	6	-	-	-
	<2.3	5			
Hb, g/dL	≥10.1	6	3.104	0.509–18.940	0.220
	<10.1	5			
WBC, 10^3^/μL	<17.0	5	-	-	-
	≥17.0	6			
CRP, mg/dL	<21.8	5	6.468	0.698–59.910	0.100
	≥21.8	6			
Predicted VC, %	≥101.7	5	6.468	0.698–59.910	0.100
	<101.7	6			
FEV_1_/FVC, %	≥66.5	5	0.580	0.097–3.479	0.551
	<66.5	6			
PS	<2	7	2.940	0.484–17.850	0.241
	≥2	4			
CCI	<3	9	0.729	0.080–6.608	0.779
	≥3	2			
Empyema	-	7	2.682	0.439–16.400	0.286
	+	4			
Tumor size, mm	<55	4	4.395	0.475–40.620	0.192
	≥55	7			
Pathological stage	II	6	2.498	0.407–15.330	0.323
	III-IV	5			
Histology	ADC	5	2.333	0.374–14.540	0.364
	Other	6			
Surgical procedure	V	1	-	-	-
	O	10			
Surgery	Lobectomy	9	6.000	0.365–98.720	0.210
	Other	2			
CR	-	7	-	-	-
	+	4			
OT, mins	<346	5	5.037	0.554–45.800	0.151
	≥346	6			
Bleeding, mL	<1327	5	5.037	0.554–45.800	0.151
	≥1327	6			

*Abbreviations*: *ADC* adenocarcinoma, *Alb* albumin, *BMI* body mass index, *CCI* Charlson comorbidity index, *CI* confidence interval, *CR* combined resection, *CRP* C-reactive protein, *F* female, *FEV*_*1*_ forced expiratory volume in 1 s, *FVC* forced vital capacity, *Hb* haemoglobin, *HR* hazard ratio, *M* male, *O* open thoracotomy, *OT* operative time, *PS* performance status, *V* video-assisted thoracoscopic surgery, *VC* vital capacity, *WBC* white blood cell

-, neither group met for the requirements for the proper statistical conditions

## Case report

**Fig. 2:**
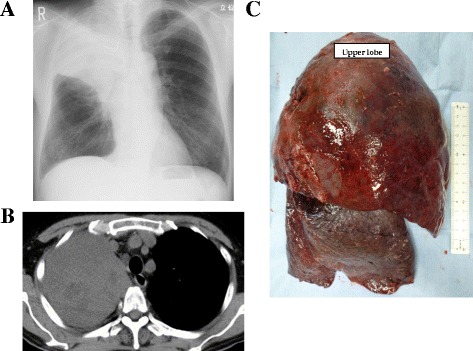
**a** Chest radiography demonstrating consolidation and atelectasis in the right upper lung field. **b** Computed tomography demonstrating necrotic cavity lesion in the right upper lobe. **c** The specimen showing the swollen right upper lobe with an intratumoral lung abscess

We present the case of a patient with intratumoral lung abscesses who was referred to our hospital for pulmonary resection (patient no. 9). The patient was a 64-year-old man who had received chemotherapy for squamous cell lung cancer in the right upper lobe (pT3N2M1a Stage IV). He was admitted to our hospital because of persistent fever. Chest radiography showed consolidation and atelectasis in the right upper lung field (Fig. 2a). He was diagnosed with post-obstructive pneumonia in the right upper lobe. Although he received antibacterial therapy for four days, his condition worsened and computed tomography showed a necrotic cavity lesion in the right upper lobe (Fig. 2b). Accordingly, right pneumonectomy was performed to remove the infection focus and improve his condition. Severe adhesions were observed over the cranial surface of the lung and chest wall as a result of infection. Additionally, his right upper lobe was swollen with an intratumoral lung abscess (Fig. 2c). The patient’s postoperative course was uneventful. He was discharged from the hospital two weeks after his operation and received definitive chemoradiotherapy. A year later, he remains alive, lives independently, and has neither had any recurrence of respiratory infections, nor progression of lung cancer.

## Discussion

Intratumoral lung abscess is considered a fatal condition and surgical procedures for intratumoral lung abscesses have sometimes been performed. Our retrospective study demonstrates that surgical procedures for advanced-stage NSCLC patients with intratumoral lung abscesses led to satisfactory post-operative mortality rates (0.0%) and acceptable prognoses, although the post-operative morbidity rate was a little high. No pre-operative, intra-operative, or post-operative prognostic factors were identified in our univariate analyses.

Davis et al. [7] reported intratumoral lung abscesses to be secondary lung abscesses and, although rare, they are potentially refractory and serious. Ishida et al. [5] reported cases of intratumoral lung abscesses after bronchoscopy and this was considered the main reason for the development of intratumoral lung abscesses. In the present study, 8 intratumoral lung abscesses were detected after bronchoscopic biopsy. Other reasons included bronchial obstruction due to malignancy [7], biopsy without bronchoscopy of the tumor, or spontaneous lung abscess in large tumor.

Lung abscesses are necrotic cavity lesions of >20 mm in diameter that contain debris and fluid [9]. Despite advances in the understanding of its microbiology and the introduction of effective antibacterial therapy, lung abscess remains a condition that is associated with significant morbidity and mortality rates [7]. Currently, the mortality rate from lung abscesses has been reported to be approximately 4–5% [10, 11]. Therefore, surgical procedures, although high-risk, have sometimes been performed to improve the clinical course of lung cancer patients with intratumoral lung abscesses [7]. Consequently, in this study, satisfactory surgical and survival outcomes were obtained, although more than half of the patients had an intraoperative blood loss of more than 1000 mL (one patient even more than 3000 mL), because of severe adhesions and long operative time.

The post-operative morbidity rate in the current study (63.6%) was higher than that of lung cancer patients with respiratory infections such as post-obstructive pneumonia (34.2%) [8]. However, considering the risk and difficulty of the surgical procedures for lung cancer patients with intratumoral lung abscesses, the fact that there was no recurrence of respiratory infections, including lung abscesses, and the post-operative mortality rate was 0.0%, this result was seemingly justified. In a Japanese Lung Cancer Registry study [12], the 5-year OS rate was 46.8% for NSCLC patients of all stages. Therefore, the 5-year OS rate of the present study (43.3%) was acceptable considering the fatal clinical course of intratumoral lung abscesses, the high-risk surgical procedures involved, and the fact that the lung cancer was higher than Stage II in all patients. In the univariate analyses, no pre-operative, intra-operative, or post-operative prognostic factors were identified, although Haraguchi et al. [8] reported low serum albumin levels and haemoglobin levels, and high pre-operative PS to be poor prognostic factors for lung cancer patients with respiratory infections such as post-obstructive pneumonia. One possible explanation for this result is that the condition of the patients in the current study was originally poorer, with lower serum albumin levels and haemoglobin levels, although pre-operative PS was lower (there were a few PS 3 patients), in comparison to patients with post-obstructive pneumonia. According to these findings, we might positively consider surgery for lung cancer with intratumoral lung abscesses if the patients are eligible for surgery.

This study was associated with some limitations. First, the sample size was small because the data were collected from a single institution. Second, the retrospective design of the study, and third, we analysed only patients who had undergone surgery and did not evaluate patients who had not undergone surgery. Third, the number of patients in this study was insufficient and the cohort of patients was highly heterogeneous to perform univariate analyses. Therefore, the results of univariate analyses might be unreliable. Further prospective studies with a larger number of patients from multiple institutions are required to confirm the findings of our present study.

## Conclusions

This study is the first to evaluate the post-operative prognoses of NSCLC patients with intratumoral lung abscesses. Surgical procedures for NSCLC with intratumoral lung abscesses, although high-risk, led to satisfactory post-operative mortality rates and acceptable prognoses in patients with advanced-stage NSCLC.

## Abbreviations

FEV_1_: Forced expiratory volume in 1 s
NSCLC: Non-small cell lung cancer
OS: Overall survival
PS: Performance status.
